# Combining *in vivo* and *in vitro* studies to elucidate the inhibitory effect of resveratrol on vorolanib metabolism

**DOI:** 10.3389/fphar.2026.1819774

**Published:** 2026-05-20

**Authors:** Dongxin Chen, Hailun Xia, Lu Cao, Haoxin Fu, Yan Chen, Shiqi Jiang, Runjuan Zhan

**Affiliations:** 1 Department of Pharmacy, The Affiliated Lihuili Hospital of Ningbo University, Ningbo, Zhejiang, China; 2 Department of Pharmacy, The First Affiliated Hospital of Wenzhou Medical University, Wenzhou, Zhejiang, China; 3 Zhejiang Key Laboratory of Intelligent Cancer Biomarker Discovery and Translation, First Affiliated Hospital, Wenzhou Medical University, Wenzhou, China

**Keywords:** combination therapy, drug-drug interactions, pharmacokinetic, resveratrol, vorolanib

## Abstract

**Introduction:**

Vorolanib is a new-generation multi-targeted kinase inhibitor (MTKI) whose drug-drug interaction (DDI) profile remains largely unexplored. This study combined *in vitro* and *in vivo* approaches to systematically investigate the effect of resveratrol on vorolanib metabolism.

**Methods:**

Using ultra-performance liquid chromatography-tandem mass spectrometry (UPLC-MS/MS), the concentrations of vorolanib and its primary active metabolite X297 were quantified in rat liver microsomes (RLM), human liver microsomes (HLM), recombinant human CYP3A4 (rCYP3A4), and Sprague-Dawley rats. Molecular docking simulations were performed to investigate the binding poses of vorolanib and resveratrol within the active site of CYP3A4.

**Results:**

*In vitro* assays revealed that resveratrol inhibited vorolanib metabolism with half-maximal inhibitory concentration (IC_50_) values of 6.28 ± 0.27, 33.86 ± 0.65 and 6.70 ± 0.32 μM in RLM, HLM and rCYP3A4, respectively. The specific mechanisms were identified as non-competitive inhibition in HLM, and mixed inhibition in both RLM and rCYP3A4. IC_50_ shift experiments indicated that the inhibition of vorolanib by resveratrol was non-time-dependent. Furthermore, metabolic stability assays in RLM showed that resveratrol substantially prolonged the *in vitro* t_1/2_ of vorolanib from 39.02 ± 2.02 to 186.24 ± 58.99 min. Molecular docking confirmed that both compounds bind within the catalytic pocket of CYP3A4 with binding energies of −7.29 and −7.23 kcal/mol, respectively. The *in vivo* pharmacokinetic study demonstrated that co-administration with resveratrol significantly increased the AUC_(0-t)_ and ${\text{AUC}}_{\left(0‐\infty \right)}$ of vorolanib by 1.70- and 1.82-fold, respectively, while significantly decreasing its CL_z/F_ by 56.1%. Similarly, for the metabolite X297, the AUC_(0-t)_, ${\text{AUC}}_{\left(0‐\infty \right)}$ , and C_max_ were increased by 2.00-, 2.22-, and 1.18-fold, respectively, accompanied by a 63.9% decrease in CL_z/F_.

**Conclusion:**

Both *in vitro* and *in vivo* results consistently demonstrate that resveratrol profoundly inhibits the metabolism and elimination of vorolanib, driven by its interference with the CYP3A4 catalytic domain as corroborated by docking analysis. These findings highlight a potential risk of herb-drug interactions, suggesting that close clinical monitoring for adverse reactions is required when vorolanib and resveratrol are used in combination.

## 1 Introduction

Vorolanib is a new-generation multi-targeted kinase inhibitor (MTKI) with a novel chemical structure (Pedersen et al., 2021). In combination with everolimus, it is indicated for patients with advanced renal cell carcinoma who have failed prior tyrosine kinase inhibitor therapy. By inhibiting tumor angiogenesis and growth, it also shows potential for the treatment of various other cancers (Pedersen et al., 2021). Vorolanib significantly affects multiple targets, including VEGFR, PDGFR, c-Kit, Flt-3, and CSF1R, and its potency is dose-dependent (Liang et al., 2022; Song et al., 2021). Compared to sunitinib, another MTKI, vorolanib exhibits a lower potential for toxicity, along with stronger kinase binding and enhanced selectivity (Liang et al., 2022). A multicenter phase III clinical study in patients with metastatic renal cell carcinoma demonstrated that the combination of vorolanib and everolimus achieved superior objective response rates and progression-free survival compared with everolimus alone (Sheng et al., 2023). Given its efficacy and safety profile in inhibiting tumor angiogenesis, vorolanib represents a promising candidate for combination therapy. However, similar to other MTKIs (e.g., sunitinib), the incidence and severity of adverse reactions rise with higher systemic exposure to vorolanib (Bagegni et al., 2022; Sheng et al., 2020). Vorolanib is primarily present in plasma in its parent form and is metabolized by CYP3A4, CYP2D6, and CYP2C9, with its primary active metabolite being the demethylated product X297. In rats, systemic exposure to X297 accounts for approximately 23.5% of that of vorolanib (Zheng et al., 2021).

Because vorolanib undergoes extensive cytochrome P450 (CYP450)-mediated metabolism, evaluating its drug-drug interaction (DDI) potential is clinically imperative (Guengerich, 2024; Manikandan and Nagini, 2018). Cancer patients frequently require polypharmacy to alleviate disease progression or comorbidities, which inherently increases the risk of DDIs (Ferri et al., 2022; Fogli et al., 2021; Raoul et al., 2023). Co-administered drugs with strong inhibitory effects on CYP450 enzymes can delay metabolism and impair drug elimination, thereby increasing systemic exposure and the risk of adverse reactions. While CYP450-mediated DDIs are a well-recognized cause of treatment discontinuation, clinical evaluation of these risks for newly approved agents remains limited. Preclinical studies using *in vitro* and *in vivo* models can therefore provide valuable predictive insights, particularly for complementary medicines such as traditional Chinese medicine (TCM), which are widely used but seldom investigated systematically (Pan et al., 2022).

TCM contains numerous bioactive compounds with well-defined structures and pharmacological activities (Yang et al., 2014). However, their influence on the metabolism of emerging targeted agents like vorolanib remains insufficiently explored. Resveratrol is a non-flavonoid polyphenol widely distributed in over 70 plant species, including *Polygonum cuspidatum*, *Mori Fructus*, grapes, and peanuts (Augimeri et al., 2021; Ren et al., 2021). Resveratrol exhibits numerous health-promoting properties, including antioxidant, anti-inflammatory, anti-allergic, and anti-cancer activities, thus attracting widespread attention as a dietary supplement and adjunctive therapy (Faisal et al., 2024). Crucially, resveratrol has been shown to significantly inhibit CYP450 enzymes, thereby altering the pharmacokinetics of co-administered drugs (Detampel et al., 2012). However, due to extensive first-pass metabolism by CYP3A4 in the intestine and liver, resveratrol has a bioavailability of less than 1%, which is further extended by the enterohepatic recirculation of its glucuronide and sulfate metabolites (Yang et al., 2025). Studies have shown that steroid sulfatase deconjugates resveratrol sulfates into free resveratrol both *in vitro* and *in vivo*, enabling resveratrol sulfates to serve as an intracellular reservoir for resveratrol (Andreadi et al., 2014). Clinical and rat trials have found that administration of resveratrol increases the area under curve of several drugs. For instance, it inhibited the metabolism of tofacitinib and erlotinib, leading to increased systemic exposure (Ye et al., 2023; Zayed et al., 2023). It has also been reported to impair the *in vitro* and *in vivo* elimination of famitinib, another MTKI (Xia et al., 2025). Given that vorolanib and famitinib share similar anti-angiogenic mechanisms and therapeutic contexts, it is critical to clarify whether resveratrol modulates vorolanib metabolism, which could pose severe DDI risks during combination therapy.

To address this knowledge gap, the present study systematically investigated the inhibitory effects of resveratrol on vorolanib metabolism. First, we established and validated an ultra-performance liquid chromatography-tandem mass spectrometry (UPLC-MS/MS) analytical method to quantify the concentrations of vorolanib and its metabolite, adapting previously established protocols (Zheng et al., 2021). Subsequently, using a constructed *in vitro* vorolanib incubation system, we conducted a preliminary DDI risk screening of 45 commonly used Chinese herbal compounds. Based on the robust inhibitory signals, resveratrol was selected for an in-depth mechanistic evaluation in rat liver microsomes (RLM) and human liver microsomes (HLM). We comprehensively assessed its impact through the determination of half-maximal inhibitory concentration (IC_50_) values, IC_50_ shifts, metabolic stability, and specific enzyme inhibition mechanisms. Furthermore, to definitively elucidate the direct enzyme-inhibitor interaction, we validated its precise specific inhibitory mechanism utilizing recombinant human CYP3A4 (rCYP3A4). Additionally, molecular docking simulations were employed to further investigate the interaction of vorolanib and resveratrol with CYP3A4. Finally, we conducted *in vivo* pharmacokinetic studies in Sprague-Dawley rats to corroborate the physiological relevance of these metabolic alterations. The comprehensive findings of this study aim to provide essential preclinical evidence to guide the safe clinical co-administration of vorolanib and resveratrol.

## 2 Methods

### 2.1 Chemicals and reagents

**FIGURE 1:**
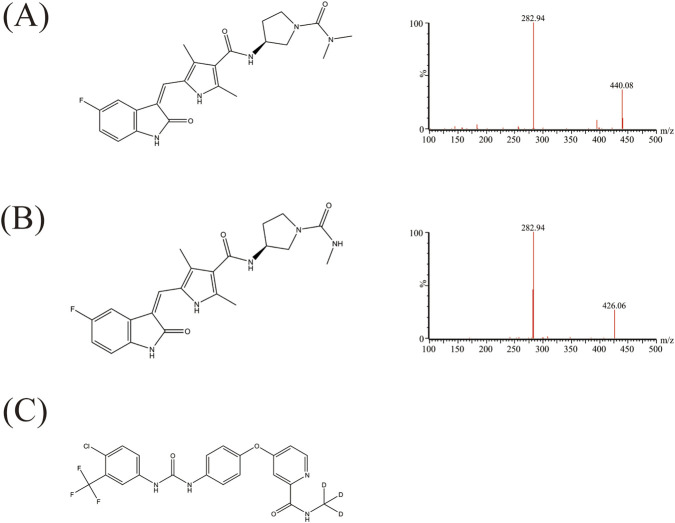
Chemical structures of vorolanib **(A)**, the metabolite X297 **(B)**, and donafenib (**(C)**, IS).

Vorolanib (Figure 1A) and its metabolite X297 (Figure 1B) were provided by Betta Pharmaceuticals Co. Ltd. (Hangzhou, China). Donafenib (Figure 1C) was used as an internal standard (IS), and 45 Chinese herbal medicine compounds, including resveratrol, were provided by Shanghai Canspec Scientific Instruments Co., Ltd. (Shanghai, China). Detailed information on these Chinese herbal medicine compounds could be found in Supplementary Table S1. RLM was prepared and its protein concentration was determined according to previous methods (Wang et al., 2015). HLM was obtained from iPhase Pharmaceutical Services Co., Ltd. (Jiangsu, China). rCYP3A4 and cytochrome b5 were prepared as previously described (Ye et al., 2024). Shanghai Aladdin Biochemical Technology Co., Ltd. (Shanghai, China) provided nicotinamide adenine dinucleotide phosphate (NADPH). All solvents used in the experiment were provided by Merck Company (Darmstadt, Germany). The purity of all drugs was ≥98%, and all solvents were of chromatographic grade.

### 2.2 UPLC-MS/MS parameters

**FIGURE 2:**
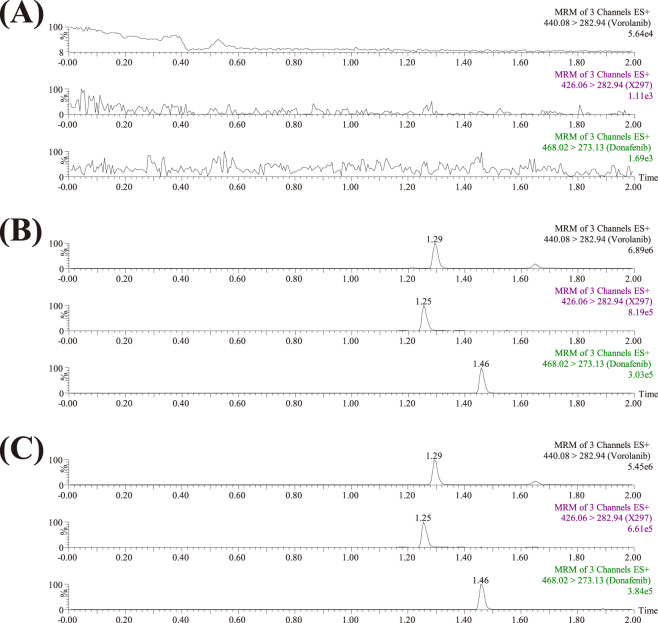
Representative MRM chromatograms of vorolanib, the metabolite X297, and donafenib (IS) in rat plasma: blank rat plasma samples (**(A)**; no analyte, no IS); blank plasma samples spiked with analytes and IS **(B)**; plasma sample collected from rats receiving a single dose of 20 mg/kg vorolanib **(C)**.

The chromatographic system utilized a Waters Acquity UPLC BEH C18 column (2.1 mm × 50 mm, particle size 1.7 μm). Gradient elution was performed at a flow rate of 0.4 mL/min for 2.0 min, consisting of 0.1% formic acid (A) and acetonitrile (B). The detailed elution program was as follows: 90% A (0-0.5 min), 90%–10% A (0.5-1.0 min), 10% A (1.0-1.4 min), 10%–90% A (1.4-1.5 min), and 90% A (1.5-2.0 min). During the analysis, the temperatures of the column and autosampler were controlled at 40 °C and 10 °C, respectively. A Waters Xevo TQS triple quadrupole mass spectrometer (Milford, MA, United States of America) operating in positive multiple reaction monitoring (MRM) mode was employed for the quantification with mass transitions of *m/z* 440.08 → 282.94 for vorolanib, *m/z* 426.06 → 282.94 for X297, and *m/z* 468.02 → 273.13 for IS, respectively. The rest of the details are shown in Table 1. As shown in Figure 2, the retention times of vorolanib, X297, and donafenib (IS) were 1.29, 1.25, and 1.46 min, respectively. No significant endogenous interference was observed at the retention times of the analytes or the IS in the blank rat plasma.

**TABLE 1:** The quantitative ion pairs and related parameters of vorolanib, the metabolite X297, and donafenib (IS).

Compound	Precursor ion (*m/z*)	Product ion (*m/z*)	Cone (V)	Collision (eV)
Vorolanib	440.08	282.94	20	11
X297	426.06	282.94	20	9
IS	468.02	273.13	30	25

### 2.3 Determination of Michaelis-Menten constant (K_m_) *in vitro* and drug screening

To determine the K_m_ of vorolanib *in vitro*, a 200 μL incubation system was first constructed. The system consisted of phosphate-buffered saline (PBS), vorolanib, and 0.3 mg/mL RLM or HLM. In the RLM incubation system, the concentrations of vorolanib were 1, 5, 10, 20, 50, 100, 200, and 300 μM. In the HLM incubation system, the concentrations of vorolanib were 1, 5, 10, 20, 50, 100, and 200 μM. Before incubation, samples were pre-incubated at 37 °C in a shaking water bath for 5 min. The reactions were initiated by the addition of 10 μL NADPH and terminated after 30 min by placing the mixtures in a −80 °C freezer. After completion, 400 μL of acetonitrile and 20 μL of IS working solution were added to each incubation system. Following complete thawing, the mixtures were vortexed for 2 min and centrifuged at 13,000 rpm at 4 °C for 10 min. Subsequently, 100 μL of the supernatant was aspirated for UPLC-MS/MS analysis to determine the concentration of the metabolite X297.

Subsequently, the inhibitory effects of 45 Chinese herbal compounds on vorolanib metabolism were investigated. These compounds are common active constituents of traditional Chinese medicines with well-documented pharmacological activities, indicating a high potential for clinical co-administration with vorolanib. To efficiently identify potent inhibitors for subsequent in-depth evaluation, a preliminary screening was conducted using a single, high inhibitor concentration of 100 μM in the previously established incubation system. The concentration of vorolanib was determined based on the K_m_ value in RLM. The remaining incubation and treatment processes were consistent with those used for determining the *in vitro* K_m_ value. Finally, the concentrations were determined using UPLC-MS/MS. Unless indicated otherwise, all subsequent processing methods were carried out in accordance with this approach.

### 2.4 Study on the effect of resveratrol on the *in vitro* metabolic inhibition of vorolanib and its mechanism

In previous studies, we observed that resveratrol exhibited a significant inhibitory effect on the MTKI famitinib (Xia et al., 2025). To determine the inhibitory effect of resveratrol on vorolanib metabolism, the subsequent experiments were conducted in both RLM and HLM to obtain the corresponding IC_50_. The concentrations in the incubation system were determined based on the K_m_ values of vorolanib in RLM and HLM. The concentrations of resveratrol were set at 0, 0.01, 0.1, 1, 10, 25, 50, and 100 μM.

To determine the effect of resveratrol on the metabolic stability of vorolanib, a 200 μL incubation system was prepared containing PBS, 1 μM vorolanib, 0.3 mg/mL RLM, and resveratrol (concentration based on its IC_50_), or a blank control. After pre-incubation for 5 min, 10 μL of NADPH was added to initiate the reaction. Incubation samples were removed at 0, 10, 20, 30, 40, 50, 60, 80, and 100 min after the reaction began and stored at −80 °C. Subsequent processing procedures were consistent with those for determining the *in vitro* K_m_ value.

To determine whether the effect of resveratrol on the metabolic inhibition of vorolanib is time-dependent, an IC_50_ shift experiment was conducted. In a 200 μL incubation system containing PBS, 0.3 mg/mL RLM, resveratrol, and with or without NADPH, the concentration of vorolanib was determined based on the K_m_. In addition, the concentrations of resveratrol were 0, 0.01, 0.1, 1, 10, 25, 50, and 100 μM. Prior to adding vorolanib to initiate the reaction, the mixture was pre-incubated at 37 °C for 30 min. Vorolanib and NADPH were then added, and the mixture was incubated for an additional 30 min.

To determine the inhibitory mechanism of resveratrol on vorolanib metabolism, an incubation system of 200 μL was established, containing PBS, vorolanib, resveratrol, and 0.3 mg/mL RLM or HLM. The concentrations of vorolanib were 0.25 K_m_, 0.5 K_m_, K_m_, and 2 K_m_ (5.08, 10.15, 20.3, and 40.6 μM for RLM and 3.87, 7.74, 15.47, and 30.94 μM for HLM), while the concentrations of resveratrol were 0, 0.25 IC_50_, 0.5 IC_50_, IC_50_, and 2 IC_50_ (0, 1.57, 3.14, 6.28, and 12.56 μM for RLM and 0, 8.47, 16.93, 33.86, and 67.72 μM for HLM). The ingredients were mixed and pre-warmed at 37 °C for 5 min. The reaction was initiated with NADPH and incubated at 37 °C for 30 min. In order to estimate the inhibition properties, Lineweaver-Burk plots were plotted and kinetic parameters were obtained. In the Lineweaver-Burk plots, the specific type of reversible enzyme inhibition was determined based on the intersection patterns of the regression lines at varying inhibitor concentrations. Specifically, competitive inhibition is indicated when the lines intersect exactly on the y-axis. Conversely, an intersection point on the x-axis denotes noncompetitive inhibition. Uncompetitive inhibition is identified when the lines are strictly parallel to each other with no intersection point. Finally, if the lines intersect within the second or third quadrant rather than on the coordinate axes, the mechanism is classified as mixed inhibition.

### 2.5 Further investigation of the effect of resveratrol on the metabolism of vorolanib in rCYP3A4

Given that vorolanib is predominantly metabolized by CYP3A4, further investigations were conducted using rCYP3A4. To determine the K_m_ of vorolanib in rCYP3A4, the concentrations of vorolanib were set at 1, 5, 10, 20, 50, 100, and 200 μM. The incubation system was contained of 0.4 pmol rCYP3A4 and 10 μg cytochrome b5. The remaining incubation conditions and experimental procedures were identical to those described in Section 2.3 for K_m_ determination.

Subsequently, the inhibitory effect of resveratrol on vorolanib metabolism in rCYP3A4 was evaluated. Based on the determined K_m_ value, the concentration of vorolanib was fixed at 5.19 μM. The remaining conditions and procedures were consistent with the methodology for IC_50_ determination detailed in Section 2.4.

Finally, to elucidate the specific inhibitory mechanism of resveratrol on vorolanib metabolism in rCYP3A4, the concentrations of resveratrol were set at 0, 0.5 IC_50_, IC_50_, and 2 IC_50_ (0, 3.35, 6.70, and 13.40 μM), while the concentrations of vorolanib were set at 0.25 K_m_, 0.5 K_m_, K_m_, and 2 K_m_ (1.30, 2.60, 5.19, and 10.38 μM). All other experimental conditions and data processing procedures were consistent with those employed for mechanism determination in Section 2.4.

### 2.6 Molecular docking

The three-dimensional structures of vorolanib and resveratrol were obtained from PubChem and used for docking. The crystal structure of human CYP3A4 (PDB ID: 5ET8) was retrieved from the Protein Data Bank. Water molecules, ligands, and other heteroatoms were removed using PyMOL. AutoDock Tools 1.5.7 was used for the pretreatment of target proteins and ligands, including the addition of polar hydrogens and the calculation of Gasteiger charges. Molecular docking was performed with AutoDock Tools using a grid box centered on the active site of CYP3A4 with dimensions of 40 Å × 40 Å × 40 Å. The binding affinity of each compound was evaluated, and the complexes with the lowest binding energies were visualized using PyMOL to analyze protein–ligand interactions.

### 2.7 Pharmacokinetics of vorolanib in rats when used in combination with resveratrol

The Institutional Animal Care and Use Committee of The First Affiliated Hospital of Wenzhou Medical University (Zhejiang, China) reviewed and approved this animal experiment (WYYY-IACUC-AEC-2025-052). Ten male Sprague-Dawley rats (200 ± 10 g) were housed under standard conditions for 7 days, with a 12-h light-dark cycle and unrestricted access to water and food. Prior to the experiment, the rats were randomly divided into two groups (control group and experimental group) and fasted for 12 h. The dosage of vorolanib was calculated based on body surface area and determined to be 20 mg/kg. The dosage of resveratrol was determined to be 50 mg/kg based on the previous literature (Singh and Bodakhe, 2022). All drugs were dissolved in corn oil and administered via gastric lavage. The experimental group received resveratrol, while the control group received the same volume of corn oil. Thirty min after resveratrol administration, vorolanib was administered to both the control and experimental groups. Blood samples were collected via the tail vein at 0.5, 1, 2, 4, 5, 6, 8, 12, and 24 h post-administration. The blood samples were centrifuged at 8,000 rpm at 4 °C for 10 min, and 100 μL of the supernatant was collected and stored at −80 °C. After all samples were collected, they were completely thawed at room temperature, and 300 μL of acetonitrile and 10 μL of the IS working solution were added. The samples were thoroughly mixed by vortexing and then centrifuged at 13,000 rpm at 4 °C for 10 min. A 100 μL aliquot of the supernatant was collected for analysis. Finally, the concentrations of vorolanib and its metabolite X297 were determined.

### 2.8 Statistical analysis

The obtained data, including K_m_, IC_50_, metabolic stability, inhibition mechanism experiments, and mean plasma concentration-time curves, were processed using GraphPad Prism 9.0 (GraphPad Software Inc., US). Non-compartmental analysis was performed using Drugs and Statistics (DAS) software (version 3.0, Shanghai University of Traditional Chinese Medicine, China) to obtain the pharmacokinetic parameters. For *in vivo* pharmacokinetic parameters, independent samples *t*-tests were conducted using SPSS 27.0 software (SPSS Inc., Chicago, Illinois, United States) to statistically investigate the differences of the parameters between the experimental and the control groups. Statistical results showed that all data were expressed as mean ± standard deviation (SD), and a *p-*value <0.05 indicated a statistically significant difference compared to the control group.

## 3 Results

### 3.1 The effects of 45 Chinese herbal compounds on the metabolism of vorolanib

**FIGURE 3:**
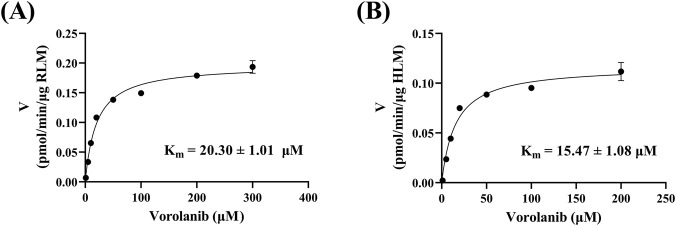
Michaelis-Menten curves of vorolanib in RLM **(A)** and HLM **(B)**. Data are presented as mean ± SD. n = 3.

**FIGURE 4:**
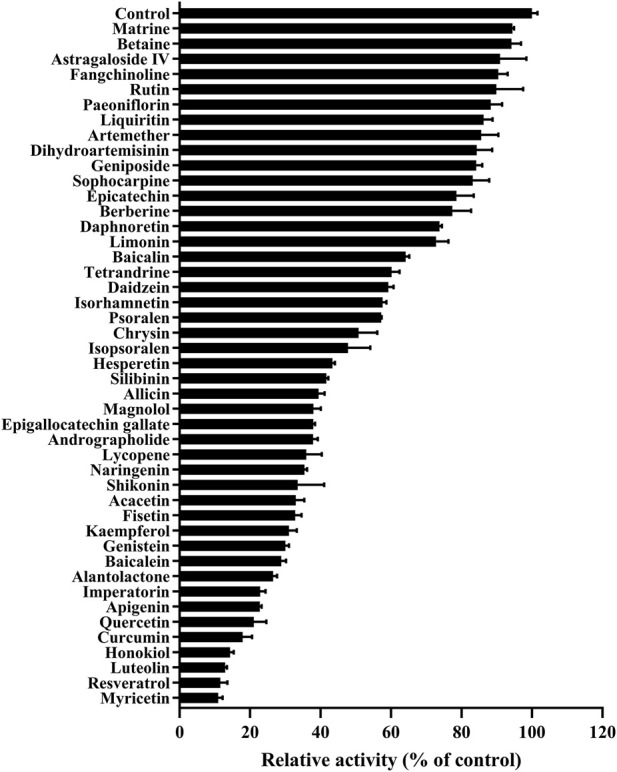
The effect of 45 Chinese herbal compounds on vorolanib metabolism. Data are presented as mean ± SD. n = 2.

As shown in Figure 3, the K_m_ values of vorolanib in RLM and HLM were 20.30 ± 1.01 and 15.47 ± 1.08 μM, respectively. Figure 4 shows the inhibitory effects of the 45 selected Chinese herbal compounds on vorolanib metabolism. Detailed inhibitory abilities can be found in Supplementary Table S1. In the presence of resveratrol, the relative activity of vorolanib metabolism in RLM (as measured by the amount of the metabolite X297 produced) was 11.60%. Additionally, myricetin, luteolin, honokiol, and curcumin reduced the relative metabolic activity of vorolanib in RLM to 10.99%, 11.60%, 14.36%, and 17.90%, respectively. A lower relative activity of the enzyme indicates that the inhibitory drug has a stronger ability to inhibit the metabolism. The relative activities of the remaining Chinese herbal compounds were between 20% and 100%, indicating they had weaker metabolic inhibitory capabilities.

### 3.2 The effect of resveratrol on the metabolism of vorolanib *in vitro* and its mechanism

**FIGURE 5:**
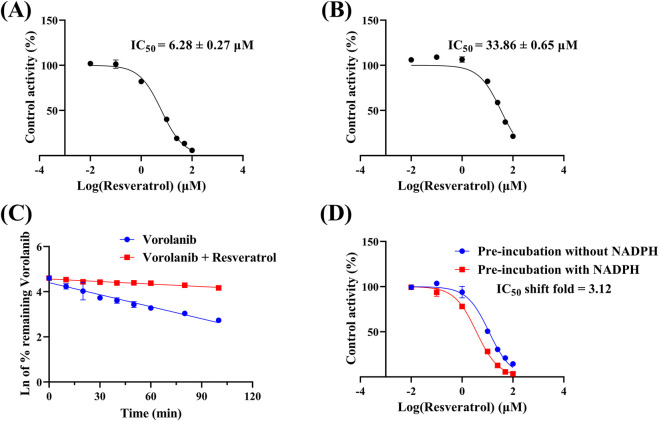
IC_50_ curves of resveratrol on vorolanib metabolism in RLM **(A)** and HLM **(B)**. The effect of resveratrol on the metabolic stability of vorolanib **(C)**. IC_50_ shift curve of resveratrol on vorolanib metabolism **(D)**. Data are presented as mean ± SD. n = 3.

**FIGURE 6:**
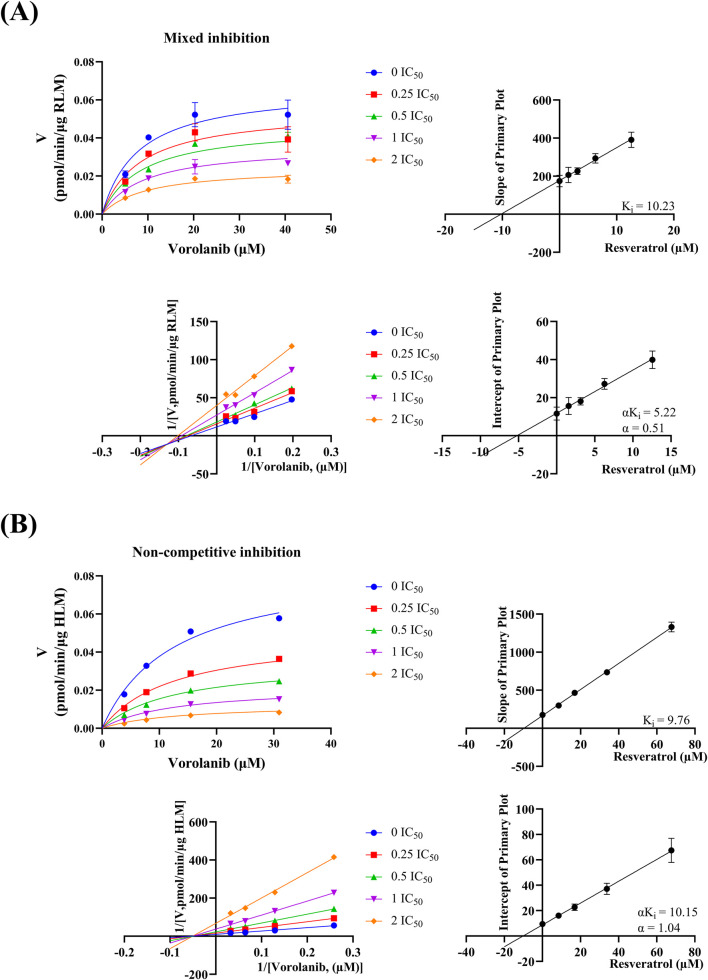
Lineweaver-Burk plots and their corresponding secondary plots for determining the inhibition constants (K_i_ and αK_i_) of resveratrol on vorolanib metabolism, revealing a mixed inhibition mechanism in RLMs **(A)** and a non-competitive inhibition mechanism in HLMs **(B)**. Data are presented as mean ± SD. n = 3.

As shown in Figures 5A,B and Table 2, the IC_50_ values of resveratrol for vorolanib metabolism in RLM and HLM were 6.28 ± 0.27 and 33.86 ± 0.65 μM, respectively. This indicated that resveratrol moderately inhibited the metabolism of vorolanib. Figure 5C and Table 3 show the effect of resveratrol on the metabolic stability of vorolanib. In the absence of resveratrol, the *in vitro* t_1/2_ of vorolanib was 39.02 ± 2.02 min, and the intrinsic clearance (CL_int_) was 59.20 ± 3.15 μL/min/mg protein. Upon the addition of resveratrol, the *in vitro* t_1/2_ of vorolanib was prolonged to 186.24 ± 58.99 min, and CL_int_ was reduced to 12.40 ± 3.18 μL/min/mg protein. These results indicated that resveratrol enhanced the stability of vorolanib in the RLM enzymatic incubation system, thereby slowing its metabolism. In addition, as shown in Figure 5D, the IC_50_ shift fold was <10, indicating that resveratrol inhibited vorolanib metabolism in a non-time-dependent manner (Jin et al., 2015). Moreover, Figure 6A showed that the inhibitory mechanism of resveratrol on vorolanib metabolism in RLM was mixed inhibition, with a K_i_ value of 10.23 μM and an αK_i_ value of 5.22 μM. Finally, Figure 6B indicated that the inhibitory mechanism of resveratrol on vorolanib metabolism in HLM was non-competitive inhibition, with a K_i_ value of 9.76 μM and an αK_i_ value of 10.15 μM. The K_i_ values in RLM and HLM were similar, indicating that resveratrol exhibited comparable binding affinities to the enzymes in both species, despite the differing inhibition types. In addition, nonlinear regression analysis of the experimental data was performed using GraphPad Prism 9.0 to verify the inhibition mechanisms. The results showed that a mixed inhibition mechanism (R^2^ = 0.9115) provided the best fit for resveratrol’s effect on vorolanib in RLM, while a non-competitive inhibition mechanism (R^2^ = 0.9911) provided the best fit in HLM. These mathematical fittings are fully consistent with the visual results demonstrated by the Lineweaver-Burk plots.

**TABLE 2:** The inhibitory effect of resveratrol on vorolanib metabolism in RLM, HLM and rCYP3A4.

Incubation system	IC_50_ (μM)	Inhibition type	K_i_ (μM)	αK_i_ (μM)	α
RLM	6.28 ± 0.27	Mixed inhibition	10.23	5.22	0.51
HLM	33.86 ± 0.65	Non-competitive inhibition	9.76	10.15	1.04
rCYP3A4	6.70 ± 0.32	Mixed inhibition	12.12	8.97	0.74

**TABLE 3:** The effect of resveratrol on the metabolic stability of vorolanib in RLM.

Parameters	Vorolanib	Vorolanib + Resveratrol
Linear regression equation	Y = −0.01776X + 4.401	Y = −0.003721X + 4.562
t_1/2_ (min)	39.02 ± 2.02	186.24 ± 58.99*
CL_int_ (μL/min/mg protein)	59.20 ± 3.15	12.40 ± 3.18*

*p < 0.05, compared with control group. T_1/2_, elimination half-life; CL_int_, intrinsic clearance. Data are presented as mean ± SD. n = 3.

**FIGURE 7:**
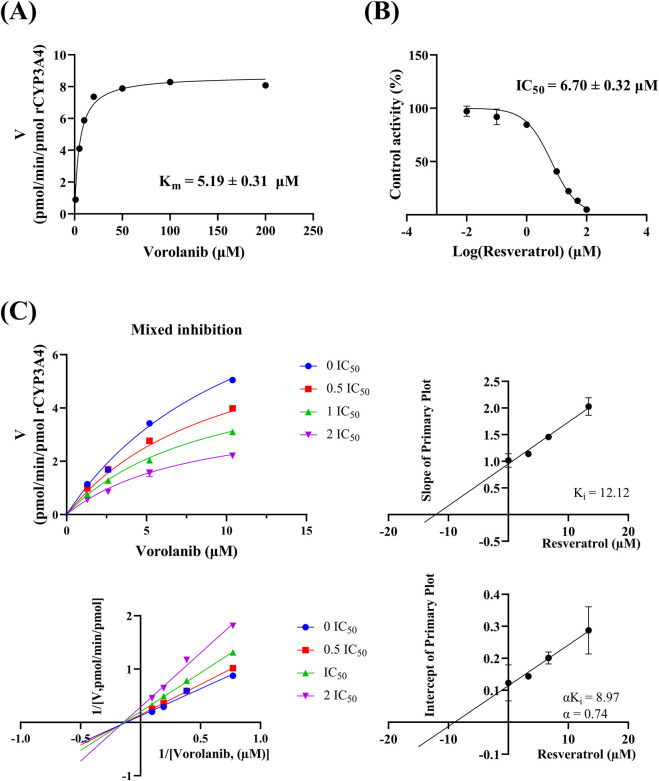
**(A)** Michaelis-Menten curve of vorolanib in rCYP3A4. **(B)** The half-maximal inhibitory concentration IC_50_ curve of resveratrol on vorolanib metabolism in rCYP3A4. **(C)** Lineweaver-Burk plots and its corresponding secondary plots for determining the K_i_ and αK_i_ of the mixed inhibition of vorolanib metabolism at varying concentrations of resveratrol. Data are presented as mean ± SD. n = 3.

In the rCYP3A4 incubation system, the K_m_ for vorolanib was determined to be 5.19 ± 0.31 μM (Figure 7A). Resveratrol exhibited a potent inhibitory effect on vorolanib metabolism, with an IC_50_ value of 6.70 ± 0.32 μM (Figure 7B). Furthermore, the Lineweaver-Burk plots revealed that the inhibition mechanism of resveratrol on vorolanib metabolism was mixed inhibition (Figure 7C). This visual determination was fully consistent with the best-fit nonlinear regression model (R^2^ = 0.9906). The specific inhibition constants, K_i_ and αK_i_, were calculated to be 12.12 and 8.97 μM, respectively. The specific kinetic parameters for the three *in vitro* incubation systems are summarized in Table 2.

### 3.3 Molecular docking simulation

**FIGURE 8:**
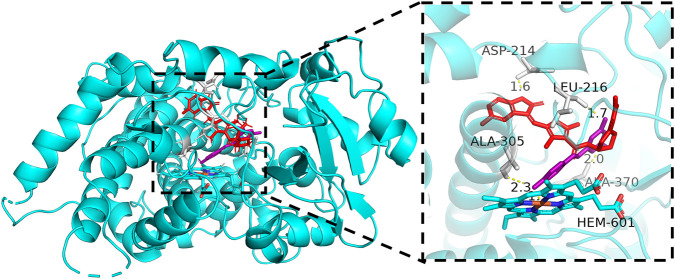
Molecular docking of vorolanib and resveratrol with CYP3A4. Superimposed 3D structure models with interactions (inset): vorolanib = red; resveratrol = purple; amino acid residues = gray; hydrogen bonding = yellow dashed line.

To gain a deeper insight into the interaction mechanism between vorolanib and resveratrol with CYP3A4, molecular docking was conducted in this study. As shown in Figure 8, both vorolanib and resveratrol successfully docked into the active site of CYP3A4 (PDB ID: 5ET8) with strong binding affinities. The binding energies for the CYP3A4-vorolanib and CYP3A4-resveratrol complexes were −7.29 kcal/mol and −7.23 kcal/mol, respectively. The binding energies were lower than −5.0 kcal/mol, indicating a strong binding affinity between vorolanib/resveratrol and the targets. Detailed interaction analysis revealed that vorolanib (red) formed hydrogen bonds (yellow dashed lines) with residues ASP-214 (1.6 Å) and ALA-370 (2.0 Å). Resveratrol (purple) formed hydrogen bonds with ALA-305 (2.3 Å) and LEU-216 (1.7 Å). The docking poses showed that both compounds were localized within the expansive catalytic cavity of the CYP3A4 enzyme.

### 3.4 The effect of resveratrol on the metabolism of vorolanib in rats

**FIGURE 9:**
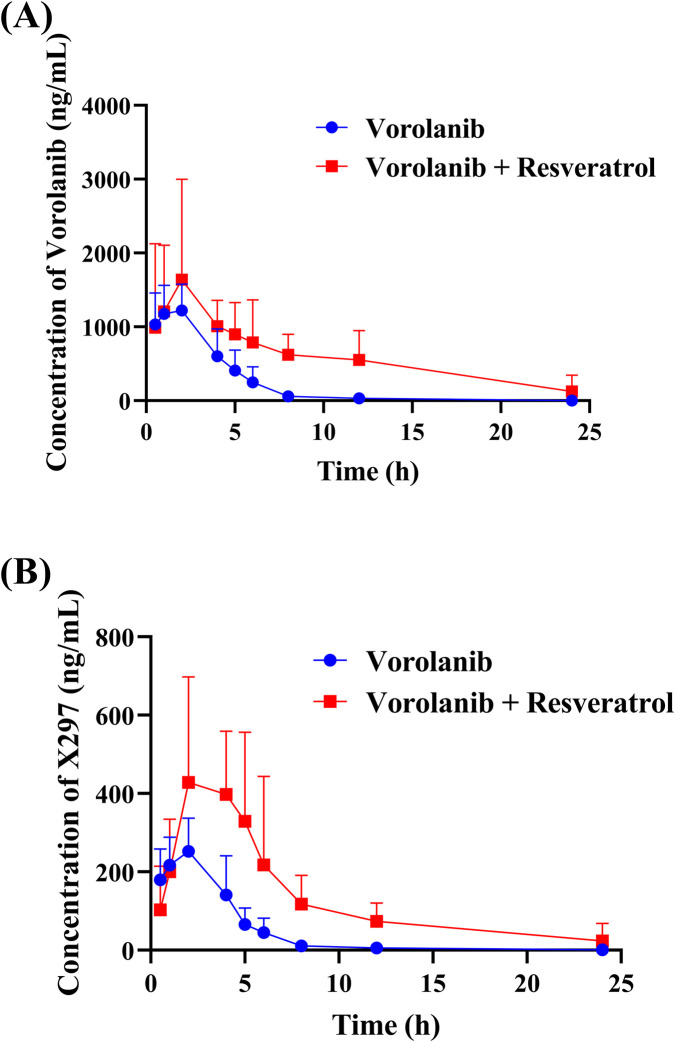
Mean plasma concentration–time curves of vorolanib **(A)** and the metabolite X297 **(B)** in Sprague-Dawley rats. Data are presented as mean ± SD. n = 5.

Figure 9 shows the mean plasma concentration-time curves of vorolanib and its metabolite X297 when vorolanib was administered alone and co-administered with resveratrol. As shown in Table 4, following co-administration with resveratrol, the main pharmacokinetic parameters of vorolanib (AUC_(0-t)_ and AUC_(0-∞)_) were significantly increased by 1.70- and 1.82-fold, respectively, while CL_z/F_ was significantly decreased by 56.1%. Additionally, as shown in Table 5, for the metabolite X297, co-administration of resveratrol with vorolanib significantly increased the AUC_(0-t)_, ${AUC}_{0-\infty }$ , and C_max_ of X297 by 2.00-, 2.22-, and 1.18-fold, respectively, while CL_z/F_ was significantly decreased by 63.9%. The results indicated that resveratrol significantly inhibited the metabolism of vorolanib and its metabolite X297 *in vivo*.

**TABLE 4:** The pharmacokinetic parameters of vorolanib in Sprague-Dawley rats.

Parameters	Vorolanib	Vorolanib + Resveratrol
AUC_(0-t)_ (ng/mL*h)	5,347.86 ± 1,133.57	14,480.45 ± 7,093.29*
AUC_(0-t)_ ratio (X297/Vorolanib)	0.20 ± 0.07	0.24 ± 0.09
${\text{AUC}}_{\left(0‐\infty \right)}$ (ng/mL*h)	5,352.23 ± 1,134.68	15,102.45 ± 7,133.31*
${\text{AUC}}_{\left(0‐\infty \right)}$ ratio (X297/Vorolanib)	0.20 ± 0.07	0.24 ± 0.09
t_1/2_ (h)	2.68 ± 0.18	4.75 ± 2.74
T_max_ (h)	1.30 ± 0.67	2.50 ± 1.50
CL_z/F_ (L/h/kg)	3.87 ± 0.81	1.70 ± 1.11**
C_max_ (ng/mL)	1,325.30 ± 358.56	2047.93 ± 1,158.08

AUC, area under the plasma concentration-time curve, t_1/2_, elimination half time, T_max_, peak time, CL_z/F_, plasma clearance, C_max_, maximum plasma concentration.

*p < 0.05, **p < 0.01, compared with the control group. Data are presented as mean ± SD. n = 5.

**TABLE 5:** The pharmacokinetic parameters of the metabolite X297 in Sprague-Dawley rats.

Parameters	Vorolanib	Vorolanib + Resveratrol
AUC_(0-t)_ (ng/mL*h)	1,058.80 ± 292.75	3,181.13 ± 1,374.58**
${\text{AUC}}_{\left(0‐\infty \right)}$ (ng/mL*h)	1,059.92 ± 291.85	3,408.11 ± 1785.96*
t_1/2_ (h)	2.61 ± 0.91	4.01 ± 1.92
T_max_ (h)	1.50 ± 0.71	3.20 ± 1.64
CL_z/F_ (L/h/kg)	19.98 ± 5.08	7.22 ± 3.62**
C_max_ (ng/mL)	269.31 ± 77.75	587.32 ± 134.88**

AUC, area under the plasma concentration-time curve, t_1/2_, elimination half time, T_max_, peak time, CL_z/F_, plasma clearance, C_max_, maximum plasma concentration.

*p < 0.05, **p < 0.01, compared with the control group. Data are presented as mean ± SD. n = 5.

## 4 Discussion

The CYP450 enzyme system is a key family of enzymes involved in drug metabolism, influencing drug plasma concentrations and clearance. For anticancer drugs, CYP450-mediated metabolism plays a crucial role in their metabolic process, directly impacting drug efficacy and safety (Mokhosoev et al., 2024). In order to improve treatment outcomes, cancer patients often choose combination therapy, with TCM being commonly used as an additional supplement to the treatment regimen (Li et al., 2025). However, many herbal components exhibit significant inhibitory effects on CYP450, potentially altering the pharmacokinetics of anticancer drugs (Hu et al., 2005). Despite the potential adverse effects of certain herbal components, patients and clinicians often continue to use such combination therapy because of its potential adjunctive therapeutic benefits. Therefore, systematic studies on drug metabolism and DDI are particularly necessary to clarify these interactions and reduce potential risks.

Vorolanib is a novel TKI that primarily targets pathways such as VEGFR and PDGFR, showing great potential in antitumor therapy (Liang et al., 2022; Song et al., 2021). Currently, basic research on vorolanib metabolism *in vitro* and *in vivo* remains limited. Existing studies have mainly focused on its clinical efficacy and safety, while its potential DDI risks have received insufficient attention. Previous studies have shown that the primary metabolic pathway of vorolanib depends on CYP3A4, indicating a significant risk of DDI when co-administered with CYP3A4 substrates, inhibitors, or inducers (Zheng et al., 2021). Meanwhile, many compounds from TCM have been identified with well-defined chemical structures and pharmacological activities that act through various signaling pathways. These active compounds are often the key substances responsible for the effects of Chinese herbal medicine. Therefore, this study selected representative Chinese herb compounds to preliminarily evaluate their potential inhibitory effects on vorolanib metabolism. Resveratrol was further selected for in-depth investigation to provide a reference for rational drug use.

At the beginning of the study, we established an *in vitro* microsomal incubation system to determine the optimal reaction rate and identify the K_m_ values. The K_m_ values in RLM and HLM were 20.30 ± 1.01 and 15.47 ± 1.08 μM, respectively, showing only a small difference between the two. We then evaluated the effects of 45 Chinese herbal medicine components on vorolanib metabolism in RLM. The results showed that most of the screened components had little effect on vorolanib metabolism. Notably, compared to the control group, the relative metabolic activities in the presence of resveratrol, myricetin, luteolin, honokiol, and curcumin were reduced to less than 20%, demonstrating significant inhibitory effects on the metabolism of vorolanib (Supplementary Table S1). Furthermore, given current laboratory resource constraints, detailed kinetic characterization (such as the determination of IC_50_) was exclusively focused on the most prominent hit, resveratrol, following the preliminary high-concentration screening. Comprehensive inhibitory risk assessments for the remaining compounds warrant further investigation in future studies. Based on our research objectives, we investigated the IC_50_ of resveratrol on vorolanib metabolism and found values of 6.28 ± 0.27 and 33.86 ± 0.65 μM in RLM and HLM, respectively. The approximately 5-fold difference in IC_50_ values highlights significant species-specific variations in hepatic metabolism. This discrepancy is primarily attributed to the inherent differences in the composition, expression levels, and catalytic site structures of CYP450 orthologs between rats and humans (e.g., human CYP3A4 versus rat CYP3A1/2) (Elsherbiny et al., 2008; Václavíková et al., 2003; Woodland et al., 2008). These structural variations in the enzyme active sites can lead to different binding affinities for both the substrate (vorolanib) and the inhibitor (resveratrol) (Khan et al., 2002). Furthermore, this marked variation underscores the necessity for caution when extrapolating *in vivo* pharmacokinetic interactions from rodent models to human clinical settings, as the inhibitory potency may be distinct in humans. *In vitro* metabolic stability experiments in RLM confirmed that resveratrol significantly affected the elimination of vorolanib, prolonging its *in vitro* t_1/2_ from 39.02 ± 2.02 min to 186.24 ± 58.99 min, while reducing CL_int_ from 59.20 ± 3.15 to 12.40 ± 3.18 μL/min/mg protein. These data indicate that resveratrol may inhibit vorolanib metabolism by modulating the activity of metabolic enzymes. Subsequently, an IC_50_ shift experiment revealed that the effect of resveratrol on vorolanib metabolism was not time-dependent. Finally, we elucidated the inhibitory mechanism of resveratrol on vorolanib. In RLM, it exhibited a mixed inhibition mechanism, whereas in HLM, it displayed a non-competitive inhibition mechanism. These differences in inhibition mechanisms may also be attributed to interspecies variations in CYP450 enzymes (Wrighton and Stevens, 1992).

To definitively clarify the precise enzymatic mechanism underlying this DDI, we further evaluated the inhibitory effect of resveratrol using rCYP3A4, the primary enzyme responsible for vorolanib metabolism. While the complex HLM system indicated a non-competitive profile, our advanced kinetic analysis in the highly purified rCYP3A4 system explicitly revealed a mixed inhibition mechanism (IC_50_ = 6.70 ± 0.32 μM, K_i_ = 12.12, αK_i_ = 8.97). This slight phenomenological variation is expected, as HLM contain a complex mixture of interplaying CYP isoforms, whereas rCYP3A4 provides an isolated environment to study direct enzyme-inhibitor interactions. Consequently, these results demonstrated that resveratrol altered the metabolism of vorolanib by specifically and potently interfering with CYP3A4.

In the present study, molecular docking simulations were performed to visualize the binding of vorolanib and resveratrol within the CYP3A4 catalytic pocket. The simulations demonstrated that both ligands could spontaneously occupy the active site near the heme iron (Figure 8). We observed that vorolanib interacted with residues ASP-214 and ALA-370, while resveratrol interacted with ALA-305 and LEU-216. Both compounds exhibited comparable binding energies (−7.29 and −7.23 kcal/mol). Importantly, their distinct interaction sites demonstrate that the expansive catalytic cavity of CYP3A4 can simultaneously accommodate both ligands. These structural findings corroborate the results from our recombinant human enzyme assays, confirming that both vorolanib and resveratrol interact with the catalytic domain of CYP3A4.

In the rat pharmacokinetic study, we observed that resveratrol significantly affected the disposition of vorolanib. Compared with the administration of vorolanib alone, co-administration with resveratrol led to significant changes in the AUC_(0-t)_, ${\text{AUC}}_{\left(0‐\infty \right)}$ , and CL_z/F_ (*p* < 0.05) of vorolanib. The significant decrease in CL_z/F_ indicates that resveratrol substantially impaired the systemic elimination of vorolanib. This *in vivo* pharmacokinetic alteration is highly consistent with our *in vitro* findings, further corroborating the potent metabolic inhibitory effect of resveratrol. For its major metabolite X297, co-administration with resveratrol also caused significant alterations in AUC_(0-t)_, ${\text{AUC}}_{\left(0‐\infty \right)}$ , CL_z/F_, and C_max_ (*p* < 0.05). Notably, although resveratrol co-administration trended towards prolonging the t_1/2_ of both vorolanib and X297, this change did not reach statistical significance (*p* > 0.05). This may be attributed to the fact that vorolanib is primarily eliminated as the parent drug, with hepatic metabolism contributing only partially to its overall clearance. Consequently, while resveratrol effectively inhibited the metabolism of vorolanib, its impact on the terminal excretion phase was relatively limited, resulting in a non-significant prolongation of the *in vivo* elimination t_1/2_. Interestingly, resveratrol did not significantly alter the metabolite-to-parent ratio despite the increased plasma exposure of both compounds. This implies that alongside hepatic metabolic inhibition, the modulation of key intestinal or hepatic transporters, including P-gp, MRP2, OAT1, and OAT3, could be an equally important mechanism driving the increased plasma exposure of vorolanib (Aires et al., 2019; Jia et al., 2016). Furthermore, according to the official prescribing information of vorolanib, caution is advised when co-administering it with P-gp modulators, strongly suggesting it acts as a P-gp substrate. Since resveratrol is a known intestinal P-gp inhibitor, it could potentially block the presystemic efflux of vorolanib, thereby enhancing its absorption. Therefore, the profound *in vivo* alterations are likely driven by a synergistic combination of CYP450 enzyme inhibition and transporter-mediated absorption enhancement. Future explicit transporter assays are required to validate this hypothesis.

While this study successfully integrated *in vitro* and *in vivo* evidence using a UPLC-MS/MS method to confirm the inhibitory effect of resveratrol on vorolanib, a limitation regarding clinical translation should be noted. Specifically, there is a notable discrepancy between the *in vitro* IC_50_ and clinically achievable human systemic plasma concentrations of resveratrol, which are typically much lower. Although this gap limits the direct extrapolation of *in vitro* parameters to human systemic interactions, our *in vivo* animal data definitively showed that resveratrol significantly elevated vorolanib exposure. This implies that presystemic inhibition in the gut and liver during the first-pass absorption remains a critical concern. Future human clinical trials and physiologically based pharmacokinetic modeling are required to bridge this gap, clarify the species-specific metabolic characteristics, and explicitly confirm the clinical relevance of this herb-drug interaction.

## 5 Conclusion

In conclusion, this study represents the first comprehensive evaluation of the inhibitory effects of 45 TCM compounds on vorolanib metabolism, uniquely elucidating the potent inhibitory mechanism of resveratrol within a systematic framework. *In vitro* studies demonstrated that resveratrol significantly inhibited vorolanib metabolism, exhibiting mixed and non-competitive inhibition mechanisms in RLM and HLM, respectively. Crucially, advanced kinetic analysis utilizing rCYP3A4 definitively confirmed a mixed inhibition mechanism, explicitly pinpointing CYP3A4 as the direct enzymatic target. Furthermore, molecular docking simulations corroborated that both vorolanib and resveratrol are capable of spontaneously binding within the catalytic pocket of CYP3A4 with comparable binding affinities. Consistent with these findings, further *in vivo* pharmacokinetic evaluations revealed that resveratrol co-administration significantly increased the systemic plasma exposure of both vorolanib and its major metabolite X297. Mechanistically, this indicates a profound herb-drug interaction driven primarily by CYP3A4-mediated hepatic metabolic inhibition, alongside potential absorption enhancement. Clinically, these findings provide critical translational evidence: the significant impact of resveratrol on vorolanib disposition highlights that stringent risk evaluation and careful therapeutic monitoring are highly warranted when these agents are co-administered in clinical settings.

## Data Availability

The original contributions presented in the study are included in the article/Supplementary Material; further inquiries may be directed to the corresponding author.
