# Supplementary material for: Combining in vivo and in vitro studies to elucidate the inhibitory effect of resveratrol on vorolanib metabolism
Source: Front Pharmacol. 2026 May 20;17:1819774. doi: 10.3389/fphar.2026.1819774 (PMC13229770; doi:10.3389/fphar.2026.1819774)
Supplement: Supplementary file 1 [file Table1.docx]

Supplementary Material

# Supplementary Table S1. Detailed information on the effects of 45 Chinese herbal medicine on vorolanib metabolism.

| **CAS** | **Name** | **Relative activity (Mean ± SD, %)** |
| --- | --- | --- |
| 529-44-2 | Myricetin | 10.99 ± 1.24 |
| 501-36-0 | Resveratrol | 11.60 ± 1.96 |
| 491-70-3 | Luteolin | 12.94 ± 0.54 |
| 35354-74-6 | Honokiol | 14.36 ± 1.00 |
| 458-37-7 | Curcumin | 17.90 ± 2.66 |
| 117-39-5 | Quercetin | 21.08 ± 3.54 |
| 520-36-5 | Apigenin | 22.77 ± 0.52 |
| 482-44-0 | Imperatorin | 22.90 ± 1.49 |
| 546-43-0 | Alantolactone | 26.53 ± 1.11 |
| 491-67-8 | Baicalein | 28.86 ± 1.35 |
| 446-72-0 | Genistein | 30.06 ± 1.00 |
| 520-18-3 | Kaempferol | 31.07 ± 2.17 |
| 528-48-3 | Fisetin | 32.81 ± 1.78 |
| 480-44-4 | Acacetin | 32.97 ± 2.44 |
| 517-89-5 | Shikonin | 33.52 ± 7.52 |
| 480-41-1 | Naringenin | 35.47 ± 0.74 |
| 502-65-8 | Lycopene | 35.95 ± 4.44 |
| 5508-58-7 | Andrographolide | 37.90 ± 1.33 |
| 989-51-5 | Epigallocatechin gallate | 37.94 ± 0.49 |
| 528-43-8 | Magnolol | 38.03 ± 2.08 |
| 539-86-6 | Allicin | 39.48 ± 1.67 |
| 22888-70-6 | Silibinin | 41.68 ± 0.56 |
| 520-33-2 | Hesperetin | 43.39 ± 0.68 |
| 523-50-2 | Isopsoralen | 47.80 ± 6.33 |
| 480-40-0 | Chrysin | 50.78 ± 5.29 |
| 66-97-7 | Psoralen | 57.22 ± 0.11 |
| 480-19-3 | Isorhamnetin | 57.61 ± 1.11 |
| 486-66-8 | Daidzein | 59.27 ± 1.46 |
| 518-34-3 | Tetrandrine | 60.17 ± 2.24 |
| 21967-41-9 | Baicalin | 64.18 ± 1.00 |
| 1180-71-8 | Limonin | 72.82 ± 3.49 |
| 2034-69-7 | Daphnoretin | 73.78 ± 0.67 |
| 2086-83-1 | Berberine | 77.44 ± 5.29 |
| 490-46-0 | Epicatechin | 78.56 ± 4.93 |
| 6483-15-4 | Sophocarpine | 83.18 ± 4.68 |
| 24512-63-8 | Geniposide | 84.22 ± 1.67 |
| 71939-50-9 | Dihydroartemisinin | 84.33 ± 4.38 |
| 71963-77-4 | Artemether | 85.62 ± 4.85 |
| 551-15-5 | Liquiritin | 86.26 ± 2.55 |
| 23180-57-6 | Paeoniflorin | 88.30 ± 3.22 |
| 153-18-4 | Rutin | 89.91 ± 7.60 |
| 436-77-1 | Fangchinoline | 90.47 ± 2.66 |
| 84687-43-4 | Astragaloside IV | 90.97 ± 7.43 |
| 107-43-7 | Betaine | 94.19 ± 2.70 |
| 519-02-8 | Matrine | 94.49 ± 0.41 |
